# Sex & menopause related differences in vascular health and rsFMRI connectivity in middle‐aged adults

**DOI:** 10.1002/alz70856_101673

**Published:** 2025-12-25

**Authors:** Julia Kearley, Bratislav Misic, Maria Natasha Rajah, Charana Rajagopal, Sophia LoParco

**Affiliations:** ^1^ Toronto Metropolitan University, Toronto, ON, Canada; ^2^ McConnell Brain Imaging Centre, Montréal Neurological Institute, McGill University, Montréal, QC, Canada; ^3^ AK, USA

## Abstract

**Background:**

Menopause is associated with hormonal changes that can impact vascular health and brain function, including resting‐state functional connectivity (rsFC). Declines in estrogen have been linked to memory disruptions and altered connectivity in networks supporting cognition^1^. Vascular risk (VR) factors, such as cholesterol, blood pressure (BP), and BMI, may exacerbate these effects, particularly in postmenopausal females^2,3^. Understanding how menopause and VR influence rsFC is crucial for identifying mechanisms underlying memory decline and informing strategies to support cognitive health in aging females.

**Method:**

We conducted **two complementary analyses** to (1) investigate how VR factors influence rsFC between **males vs. females** and (2) examine whether menopause status in middle‐aged females influences these associations. Rs‐fMRI data was collected from 42 premenopausal, 41 postmenopausal and 39 male participants. FC matrices were computed using 200 cortical ROIs^4^ and 4 hippocampal ROIs^5^. VR was assessed using measures of BMI, exercise, education, age, cholesterol, and systolic BP. VR factors were entered as behavioral vectors in two‐group B‐PLS analyses (Analysis #1: males vs. females; Analysis #2: premenopausal vs. postmenopausal females) to identify group‐specific rsFC patterns.

**Result:**

Analysis 1 identified both similarities and differences in the effect of VR on rsFC in males and females. Both sexes exhibited increased FC between hippocampus (HC) and other cortical networks, and decreased FC between Dorsal and Ventral Attention networks (DAN, VAN) for individuals with higher cholesterol, who also exercised regularly. In males this pattern was also associated with BMI. In females this pattern of FC was also associated with higher BP, and older age. In addition, females uniquely showed increased FC between DAN and Control (CON) and Default Mode (DMN) networks, respectively, which was also related to increased cholesterol, BP, age and regular exercise. Analysis 2 indicated the effects observed in females in Analysis 1 were driven by postmenopausal females.

**Conclusion:**

Sex and menopause status shape the relationship between VR and rsFC. Females appear more vulnerable to VR‐related rsFC changes, particularly postmenopausal females, where cholesterol, age, and BP affected rsFC involving the hippocampus, DMN, DAN, and Control networks. Interestingly, BMI was not associated with rsFC in females, but was in males.

